# Trauma‐related guilt, shame, and trauma type among patients with co‐occurring PTSD and SUD

**DOI:** 10.1111/acer.70028

**Published:** 2025-03-26

**Authors:** Nathalie N. M. Faber, Sera A. Lortye, Loes A. Marquenie, Anna E. Goudriaan, Arnoud Arntz, Marleen M. de Waal

**Affiliations:** ^1^ Arkin Mental Health Care, Jellinek, Amsterdam Institute for Addiction Research Amsterdam The Netherlands; ^2^ Department of Psychiatry, Amsterdam UMC University of Amsterdam Amsterdam The Netherlands; ^3^ Amsterdam Public Health Research Institute Amsterdam The Netherlands; ^4^ Department of Clinical Psychology University of Amsterdam Amsterdam The Netherlands

**Keywords:** index trauma, PTSD, SUD, trauma‐related guilt, trauma‐related shame

## Abstract

**Background:**

Substance use disorder (SUD) frequently co‐occurs with posttraumatic stress disorder (PTSD). Feelings of shame and guilt are associated with either disorder but have not been studied in patients with both disorders. Index trauma characteristics are associated with PTSD severity and trauma‐related shame. This study examines the effects of trauma‐related guilt and shame, and index trauma on SUD and PTSD severity in a clinical sample of individuals with co‐occurring SUD and PTSD.

**Methods:**

Participants were SUD‐treatment‐seeking patients with co‐occurring PTSD (*N* = 209) who completed the Clinician‐Administered PTSD scale for Diagnostic and Statistical Manual of Mental Disorders (DSM‐5; CAPS‐5), Alcohol Use Disorder Identification Test (AUDIT), Drug Use Disorders Identification Test (DUDIT), Trauma‐Related Guilt Inventory (TRGI) and Trauma‐Related Shame Inventory (TRSI). Regression analyses examined the predictive values of PTSD severity, trauma‐related guilt, and shame on alcohol and drug use problems, and the predictive values of trauma‐related guilt and shame on PTSD severity. One‐way ANOVA and follow‐up *t*‐tests examined the effects of index trauma on PTSD severity and trauma‐related shame.

**Results:**

PTSD severity was significantly associated with drug use disorder (DUD) severity and showed a curvilinear relationship to alcohol use disorder (AUD) severity. Trauma‐related guilt was not significantly associated with SUD severity, while trauma‐related shame was significantly associated with DUD severity (but not AUD severity). Both trauma‐related guilt and shame were significantly associated with PTSD severity; however, only trauma‐related shame showed an independent association. Interpersonal (especially sexual) index traumas were associated with increased trauma‐related guilt and shame, while childhood index traumas were associated with increased PTSD severity.

**Conclusions:**

Trauma‐related guilt and shame might be important focus points in PTSD treatment, but for SUD problems, this study only showed an association between trauma‐related shame and drug use problems. Trauma‐related shame seems to be a more important treatment focus point than trauma‐related guilt in the treatment of PTSD. It becomes particularly relevant for interpersonal index traumas (especially sexual trauma). Childhood traumas require attention in SUD‐PTSD co‐occurrence, given the higher severity of PTSD.

## Graphical abstract

This article analyzes baseline data from a large Dutch RCT involving 209 patients with co‐occurring PTSD and SUD. PTSD severity was linearly associated with drug use disorder (DUD) severity and curvilinearly associated with alcohol use disorder (AUD) severity. Trauma‐related guilt and shame were associated with PTSD severity, with shame showing a stronger association. For SUD, only trauma‐related shame was associated with DUD severity. Index trauma was significantly associated with trauma‐related guilt, shame, and PTSD severity. Findings have clinical and research implications.

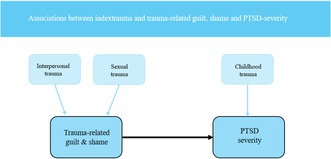

## INTRODUCTION

Substance use disorder (SUD) is a condition characterized by the recurring use of substances despite significant harmful consequences (American Psychiatric Association [APA], 2013). It frequently co‐occurs with posttraumatic stress disorder (PTSD), which can occur after experiencing or witnessing a traumatic event (Gradus, 2017; Jacobsen et al., 2001). Recent research has shown that more than half of the individuals with lifetime PTSD have had a lifetime SUD (Simpson et al., 2019), and in treatment‐seeking SUD patients, the co‐occurrence of PTSD is estimated to be just below 40% (Gielen et al., 2012). The co‐occurrence of these disorders is associated with higher symptom severity and a more complex and costlier clinical course when compared with either disorder alone (Driessen et al., 2008; Schäfer & Najavits, 2007). Individuals with both PTSD and SUD show worse treatment adherence, their symptoms improve less during treatment, and they have shorter periods of abstinence after treatment. In addition, patients show more psychiatric, medical, legal, and social problems than those with either disorder alone (Najavits et al., 2010; Roberts et al., 2015).

In recent research, focus has been put on identifying factors that are both related to PTSD and SUD, to increase the understanding of the co‐occurrence of both disorders and identify possible focus points for treatment. Proneness to feelings of guilt and shame has received considerable attention as one of such possible factors, in relation to PTSD and SUD (Luoma et al., 2019; Shi et al., 2021). Guilt and shame are both seen as social emotions involving negative affect, but with a clear distinction (for a more extensive overview on this distinction, readers are referred to Batchelder et al., 2022 or Kubany & Watson, 2003). Guilt is seen as resulting from a negative evaluation of one's specific behaviors, often motivating the individual to repair the results of their behavior. On the other hand, shame is seen as resulting from a negative evaluation of the entire self (Eaton et al., 2020; Kubany & Watson, 2003; Tangney & Dearing, 2003). Research has also shown that feelings of guilt about one's behaviors can eventually lead to feelings of shame about one's self (Norman et al., 2014). Following this distinction, shame is often seen as a more maladaptive emotion than guilt. An important addition is the distinction between general feelings of guilt or shame (proneness), or context‐dependent feelings of guilt or shame (e.g., trauma‐related feelings of guilt and shame; Rüsch et al., 2007; Tangney & Dearing, 2003).

In relation to SUD, research has consistently found a positive relationship between shame proneness and SUD severity in patients presenting with SUD (Dearing et al., 2005; Held et al., 2015; Holl et al., 2017; O'Connor et al., 1994). This is in line with the shame spiral of addiction (Batchelder et al., 2022), which posits that shame‐prone individuals use substances to dampen negative self‐conscious emotions, which paradoxically leads to increased feelings of shame related to the stigma of being a substance user. For guilt proneness and SUD, however, research does not find a positive relationship. Instead, research has shown that guilt proneness can be either unrelated to SUD severity, or negatively related (Dearing et al., 2005; Eaton et al., 2020; Held et al., 2015; O'Connor et al., 1994; Treeby & Bruno, 2012). This is in line with what was mentioned earlier about the adaptiveness of guilt. Feelings of guilt about substance use can have a protective effect against the development of problematic substance abuse patterns (Dearing et al., 2005). Regarding the difference between general proneness and context‐dependent feelings of guilt and shame, the association between trauma‐related guilt and shame and SUD has not received considerable attention in research yet. To our knowledge, only one study researched trauma‐related guilt in patients with co‐occurring PTSD and SUD and found no association between trauma‐related guilt and SUD severity (Capone et al., 2020). In addition, Saraiya et al. (2024) studied the association of trauma‐related shame with daily nonmedical prescription opioid use among individuals with clinical and subclinical PTSD symptoms. They found that trauma‐related shame significantly predicted the use of opioids over a period of 28 days, above and beyond the predictive effect of PTSD symptoms. Limitations were a small sample size (*N* = 38), the inclusion of both clinical and subclinical PTSD patients, and the focus on opioid use only. Studying the specific effect of trauma‐related guilt and shame on SUD in patients with co‐occurring PTSD and SUD could provide information on possible treatment focus points in these patients.

In relation to PTSD, studies have consistently found strong associations between feelings of guilt and shame and PTSD (e.g., Hoppen et al., 2022), resulting in the adaptation of the criteria for PTSD in the Diagnostic and Statistical Manual of Mental Disorders (DSM‐5; American Psychiatric Association [APA], 2013, Thole). Importantly, the type of measure of guilt and shame is found to be a significant moderator for the association found between both feelings and PTSD (Jones & Badour, 2024; Shi et al., 2021). Instruments measuring guilt or shame proneness or shame have shown associations with PTSD; however, instruments directly measuring trauma‐related guilt or shame show the strongest association with psychopathology and PTSD specifically (DeCou et al., 2023; Shi et al., 2021). Additionally, some studies show a suppression effect when both guilt and shame are entered into the same model, where the effect of guilt on PTSD severity is canceled out and the effect of shame on PTSD severity is strengthened (Leskela et al., 2002; Paulhus et al., 2004; Street & Arias, 2001). Recently, a meta‐analysis was published on the associations between guilt, shame, and PTSD (Shi et al., 2021). This revealed that both guilt and shame show significant, positive correlations with PTSD severity. In addition, they found that the effect size of the association between shame and PTSD was larger, with or without partialing out the effect of guilt. These findings suggest a more prominent role of shame in PTSD severity.

Certain specific characteristics of the index trauma have been studied in their association with PTSD severity and trauma‐related feelings of shame. Forbes et al. (2012) found in their longitudinal study that interpersonal traumatic experiences (physical and sexual assault) have a stronger association with PTSD severity than noninterpersonal traumatic experiences. They proposed that exposure to interpersonal traumatic experiences is more pathogenic than exposure to noninterpersonal traumatic experiences because interpersonal traumatic experiences involve a threat to the individual's assumption about the safety and predictability of significant others, in addition to the experience of a direct threat. Additionally, the perpetrator of interpersonal traumatic experiences is often a known and trusted individual for the victim, possibly leading to difficulties in their support system or perceived support (Forbes et al., 2013; Hughesdon et al., 2021). Forbes et al. (2013) also found that intimate interpersonal traumatic experiences (sexual assault or physical assault by a loved one) are more strongly associated with PTSD severity than nonintimate interpersonal traumatic experiences (physical assaults by a nonintimate perpetrator) and noninterpersonal traumatic experiences. Interpersonal traumatic experiences are also linked to more severe trauma‐related guilt and shame than noninterpersonal traumatic experiences (e.g., Baker et al., 2021). Again, studies have shown intimate interpersonal traumatic experiences (sexual trauma) to be most strongly associated with trauma‐related shame (Amstadter & Vernon, 2008; Wetterlöv et al., 2021). Another characteristic of the type of index trauma that has received a lot of attention is the age at which the victim was exposed to the traumatic experience. Several studies have found an association between childhood exposure to trauma and the severity of PTSD symptoms (e.g., Dunn et al., 2017; Miller‐Graff et al., 2016). Regarding trauma‐related feelings of guilt and shame, Hagenaars et al. (2011) found no association with childhood trauma exposure after controlling for PTSD severity.

Several studies have found an association between PTSD and SUD (e.g., Berenz et al., 2017). Trauma‐related emotions of guilt and shame could possibly contribute to this association between PTSD and SUD, but as of yet, there is limited evidence for the associations between trauma‐related guilt and shame and SUD severity. Additionally, index trauma could potentially affect the association between trauma‐related emotions and PTSD severity. Limited research has been conducted regarding the associations between PTSD severity, SUD severity, trauma‐related guilt and shame, and index trauma, especially in a clinical sample of patients presenting for SUD treatment with co‐occurring PTSD, who often are excluded from research. To that end, this study is designed to disentangle the relative effects of trauma‐related guilt and shame, and type of index trauma on SUD and PTSD severity by examining the following hypotheses in a sample of individuals with co‐occurring SUD and PTSD:

1. PTSD severity is significantly and positively related to SUD severity.
2. Trauma‐related shame is significantly and positively related to SUD severity. The association between trauma‐related guilt and SUD severity will be explored.
3. Trauma‐related shame and guilt are both significantly and positively related to PTSD severity. However, it is expected that only trauma‐related shame is independently associated with PTSD severity when both predictors are added to the same regression model.
4. It is expected that this type of index trauma is significantly associated with both trauma‐related shame and PTSD severity. More specifically,
  - Interpersonal trauma is expected to be more strongly associated with PTSD severity and trauma‐related shame than is noninterpersonal trauma.
  - Sexual interpersonal trauma is expected to be more strongly associated with trauma‐related shame than physical interpersonal trauma. Differences in PTSD severity will be researched exploratorily since we could not distinguish between physical interpersonal trauma with an intimate perpetrator or a nonintimate perpetrator in our data.
  - Childhood exposure to interpersonal trauma is expected to be more strongly associated with PTSD severity than nonchildhood exposure to interpersonal trauma. Differences in trauma‐related shame will be researched exploratorily.
  - If hypothesis 3 is confirmed and there is a significant effect of type of index trauma (a, b, or c) on PTSD severity, a moderation effect of index trauma on the association between trauma‐related shame and PTSD severity will be explored.

## METHODS

### Participants and procedures

This study used baseline data of a randomized controlled trial conducted at two departments from a Dutch SUD‐treatment center. Participants were SUD‐treatment‐seeking patients with co‐occurring PTSD. The RCT examined simultaneous versus sequential treatment of PTSD in patients with comorbid SUD and PTSD and compared three types of PTSD treatment as add‐on to SUD treatment versus SUD treatment‐only. The trial design was approved by the Medical Ethical Committee of the Amsterdam Academic Medical Centre (AMC) and was registered at the “Nederlands Trial Register” (NTR L7885). Full details about the study, including the sample size calculation, have been published in a study protocol article (Lortye et al., 2021).

Inclusion criteria were as follows: (a) age 18 years or older; (b) substance use disorder(s) according to the DSM‐5 (APA, 2013), with a primary diagnosis involving one of the following substances: alcohol, cannabis, cocaine (snorting), amphetamine, benzodiazepine, or opioid; (c) posttraumatic stress disorder according to the DSM‐5 criteria; (d) sufficient understanding of the Dutch language to be able to fill out Dutch questionnaires and follow therapy in Dutch (Lortye et al., 2021). Exclusion criteria were examined in structured inclusion interviews performed by junior researchers and read as follows: (a) acute psychotic disorder; (b) intellectual disability or cognitive impairment (estimated IQ < 70); (c) current physical or sexual abuse or death threats; (d) current acute suicidal behavior (high suicide risk and suicide attempt in the last 3 months); (e) life‐threatening self‐injury; (f) homelessness; (g) involvement in a compensation case or legal procedures concerning admission or stay in the Netherlands; (h) involvement in legal procedures regarding the index trauma; and (i) engagement in any other current PTSD treatment.

During their routine SUD intake procedure, all patients filled in the J‐PTSD: a PTSD screening instrument that was specifically designed for and validated in a SUD population (van Dam et al., 2010). Patients with a positive PTSD screener were invited for a clinician‐administered PTSD scale (CAPS‐5) interview to determine a diagnosis of PTSD (Weathers et al., 2018). Patients with a diagnosis of PTSD received information about the study and were invited for an informed consent procedure and screening of inclusion and exclusion criteria. Potential participants were invited for a baseline assessment (T0) and inclusion interview, approximately 2 weeks before the start of their SUD treatment. In total, four assessments were conducted with three follow‐up assessments at 3, 6, and 9 months after the baseline assessment. After the baseline assessment, participants were randomized for a parallel or sequential PTSD treatment and for PTSD treatment type: Prolonged Exposure (PE), Eye Movement Desensitization and Reprocessing (EMDR), or Imagery Rescripting (ImRs). The baseline assessment was conducted by a junior researcher (MSc in Psychology) and consisted of a combination of a (clinician‐administered) structured interview and self‐report questionnaires. For this article, the baseline assessment of the structured interview and five questionnaires were selected with regard to PTSD severity, type of index trauma, SUD problems, PTSD‐related shame, and PTSD‐related guilt. Some sociodemographic characteristics were included as well, such as age, sex, years of SUD problems, and years of PTSD problems.

### Measures

#### PTSD severity and index trauma type

The Clinician‐Administered PTSD Scale for DSM‐5 (CAPS‐5) was used to determine the index trauma and to determine PTSD severity. The CAPS is a 30‐items structured interview, in line with the PTSD diagnosis structure of the DSM‐5. It is the gold standard structured interview for a PTSD diagnosis. The questions target symptom onset and duration as well as subjective distress and impairment. Ratings range from 0 to 4, and by summing the scores from item 1 to 20 a total symptom severity score is calculated with a range from 0 to 80 ascending in severity. Psychometric properties for the Dutch version of the CAPS‐5 are adequate in a sample of Dutch individuals with different trauma backgrounds (Boeschoten et al., 2018; Weathers et al., 2018). In the current sample, the internal consistency of the symptom severity scale is acceptable (*α* = 0.73) (Gliem & Gliem, 2003). Based on previous research, the answers to the A‐criterion of the CAPS‐5 were divided into different categories, namely, (1) sexual assaults; (2) child sexual abuse; (3) accidents; (4) disasters; (5) nonsexual assaults; (6) nonsexual child abuse and neglect; (7) combat, war, and terrorism; (8) witnessing death and injury; and (9) illness and unspecified injury (Tolin & Foa, 2006). For patients who learned that a trauma happened to a close friend or family and other traumas that did not fulfill any of the above‐described categories, we added an extra tenth category “other A‐criterion traumas” (Kessler et al., 2017). When the index trauma was explicitly labeled as one of the nine categories, it was coded as such. For index traumas that were not explicitly labeled as one of the categories, two raters coded the category of the index trauma. Inter‐rated evaluation was needed for three participants. When information about the index trauma could be interpreted in multiple ways, further patient file study was conducted in order to categorize it. This was needed for seven participants. In cases the index trauma could be categorized in two different categories, experiencing the trauma was chosen above witnessing the trauma and sexual traumas were scored above physical traumas. Since only one person was coded into “illness & unspecified injury,” this category merged with “other A‐criterion traumas.” Since there were no persons in the category “disaster,” this category was redundant.

#### Alcohol and drug use problems

In order to assess alcohol use problems, the Dutch version of the Alcohol Use Disorder Identification Test (AUDIT) was used, whereas in order to assess drug use problems the Dutch version of the Drug Use Disorders Identification Test (DUDIT) was used. Both instruments were developed by the World Health Organization (WHO) to use as parallel instruments for the screening of alcohol and drug use problems. The AUDIT consists of 10 items screening for a range of alcohol risk behaviors, including hazardous alcohol use (items 1–3), dependence symptoms (items 4–6), and harmful alcohol use (items 7–10) in the past year. For this study, the past 3 months were queried. Each item is scored from 0 to 4, and by summing up all items a total severity score can be calculated, with higher scores indicating higher levels of alcohol use and problems related to alcohol use. Total scores of 8 and above are recommended as indicators of hazardous/harmful use of alcohol (Babor et al., 2001). A systematic review has shown that the AUDIT has good psychometric properties, measured with diverse samples and contexts (de Meneses‐Gaya et al., 2009) and more specifically for the Dutch version, the AUDIT is stated to be a reliable screening instrument (Hildebrand & Noteborn, 2015). In the current sample, the internal consistency of the scale was excellent (*α* = 0.94) (Gliem & Gliem, 2003). The DUDIT consists of 11 items in relation to drug use and drug use problems in the past year. For this study, the past 3 months were questioned. Nine items are scored on 5‐point scales ranging from 0 to 4 and two items are scored on 3‐point scales with values of 0, 2, and 4. Thus, the total severity score ranges from 0 to 44, with higher scores indicating higher levels of drug use and problems related to drug use (Hildebrand, 2015). The Dutch version of the DUDIT turns out to be a reliable screening instrument (Hildebrand & Noteborn, 2015). In the current sample, the internal consistency of the scale was excellent (*α* = 0.93) (Gliem & Gliem, 2003).

#### Shame

The Dutch version of the Trauma‐Related Shame Inventory (TRSI) was used to assess the degree of various aspects of shame related to experienced trauma. The TRSI consists of 24 items scored between 0 to 3 with higher scores indicating higher levels of trauma‐related shame. The sum of all items gives a total score with a range of 0–72, ascending in severity. For the TRSI, both a total score scale and an internal (perceptions of the self as defective) and external scale (perception of others' negative evaluation of the self) can be determined. A sample item of the internal scale is “As a result of my traumatic experience, I cannot accept myself,” and for the external scale “If others knew what happened to me, they would find me unacceptable.” For this study, we made use of the total scale of the TRSI to include both internal and external trauma‐related shame. Research showed good psychometric properties of the TRSI (Øktedalen et al., 2014). In this sample, the internal consistency for the total scale was excellent (*α* = 0.95) (Gliem & Gliem, 2003).

#### Guilt

The Dutch translation of the Trauma‐Related Guilt Inventory (TRGI) was used to assess severity of trauma‐related guilt. The TRGI consists of 32 questions regarding feelings of guilt that are related to exposure to traumatic events. Items are scored between 0 to 4, ascending in severity of trauma‐related guilt, with eight items reverse scored. The TRGI uses three subscales: Global Guilt, Guilt Distress and Guilt Cognitions. In this study, we have made use of the Guilt Cognition scale, which contains 22 items (see for comparison Resick et al., 2008; see Appendix S1 for the process of selecting this subscale). A sample item of this scale is “What I did was unforgivable.” Research showed good psychometric properties of the TRGI and of the Guilt Cognitions scale (Kubany et al., 1996). In the current sample, the internal consistency of the Guilt Cognitions scale was excellent (*α* = 0.91).

### Statistical analysis

The raw data were processed using the Statistical Package for the Social Sciences (SPSS) program, version 27 (IBM Corp Armonk, USA, 2017). For all tests, a significance level of *α* = 0.05 was used. The data were recoded where needed so that high numerical scores represent high scores on the constructs. There were no missing values on the interview and questionnaires. On the sociodemographic question regarding the duration of PTSD symptoms, there were three missing values and two missing values with regard to the duration of SUD. To the question regarding the order of the symptoms, there were three missing values.

Frequency tables were used to describe the population and the different types of index trauma and severity of all the symptoms. Furthermore, the internal consistency of all the questionnaires was calculated. Separate regression analyses were performed to examine the predictive value of PTSD severity, trauma‐related shame, and trauma‐related guilt on both alcohol and drug use problems. For analyses on alcohol use problems (AUDIT), we corrected for drug use problems (DUDIT), and for analyses on drug use problems, we corrected for alcohol use problems (see Appendix S1 on these analyses). To examine the predictive value of trauma‐related guilt and shame on PTSD severity, first a linear regression with only trauma‐related guilt as a predictor was performed; then, only trauma‐related shame was added, and in the third model, trauma‐related guilt and shame were both added as predictors. To examine the effects of index trauma on trauma‐related guilt, shame, and PTSD severity, first we explored the effects of all index trauma on these dependent variables. We performed three separate one‐way ANOVAs without collapsing the index trauma to the categories. Due to small cell sizes and the exploratory nature of these analyses, a more liberal *α* of 0.10 was used (Hsu, 1996). Then, to more specifically test the hypotheses about (sexual) interpersonal and childhood traumas, the index traumas were reduced to three comparisons: interpersonal versus noninterpersonal; sexual interpersonal versus physical interpersonal; and childhood versus nonchildhood. Independent *t*‐tests were performed to explore whether these three comparisons significantly differed for trauma‐related shame, PTSD severity, and trauma‐related guilt, using an *α* of 0.05. Finally, the PROCESS Macro was used to examine whether trauma‐related shame is a moderator of the relationship between the type of index trauma and PTSD severity.

All regression analyses were initially performed as described above, without the addition of covariates. Subsequently, we ran multiple models, including varying combinations of covariates for age, sex, location of admission, and duration of PTSD/SUD symptoms. Since none of the models, including covariates, produced different results from the initial model, we opted to report on the most parsimonious model, incorporating the fewest predictors and thus not including covariates.

Sensitivity power analyses were performed in G*Power version 3.1.9.7 (Faul et al., 2007) all indicating that the used sample sizes for each regression analysis give 80% power to detect a small effect size with a significance level of 0.05 (*f*2 = 0.05). For the ANOVA, 80% power was indicated to detect a medium to large effect (*f*2 = 0.27) and for the follow‐up analyses of hypothesis 4 (*t*‐tests), sensitivity power analyses indicated 80% power to detect small to medium effect sizes around *d* = 0.40 with a significance level of 0.05. For all analyses, the assumptions of linearity, multivariate normality, multicollinearity, and homoscedasticity were checked.

## RESULTS

A total of 209 patients were recruited, with a mean age of 37.5 years (SD = 11.99); the sample was 53.6% male and 46.4% female. Participants predominantly were in treatment for a primary diagnosis of alcohol use disorder (47.4%, *N* = 99), followed by cannabis (33.5%, *N* = 70) and cocaine (10%, *N* = 21). Multiple diagnoses of SUD were prevalent in 92 participants (44%). Most participants were single (71.7%, *N* = 150) and many were living alone (40.2%, *N* = 84). Further descriptive statistics of the study participants are presented in Table 1. Descriptive statistics of the study variables and their correlations are presented in Table 2.

**TABLE 1:** Sociodemographic sample characteristics (*N* = 209).

	*N*	%
Mean age (SE)	37.45 (11.99)	
Mean years of PTSD complaints (SE)	17.75 (13.34)	
Mean years of SUD complaints (SE)	12.21 (10.62)	
Sex		
Male	112	53.6
Female	97	46.4
Country of birth		
Netherlands	171	81.8
Other European country	9	4.3
Non‐European country	29	13.9
Country of birth parents		
Both Netherlands	116	55.5
One Netherlands, one other country	25	12
Both other countries	68	32.5
Education		
No degree, primary school, secondary school lower level	53	25.3
Secondary school, higher level	87	41.6
Postsecondary	66	31.6
Other	3	1.4
Source of income		
No paid work	74	35.4
Paid work	107	51.2
Student without work	23	11
Other	5	2.4
Primary substance use disorder (SUD)		
Alcohol	99	47.4
Cannabis	70	33.5
Cocaine	21	10
Sedating substances (e.g., benzodiazepines)	7	3.3
Other	12	5.8
Average amount of SUDs	*M* = 1.62	SD = 0.78
Index trauma		
Sexual assault	21	10
Child sexual abuse	55	26.3
Accidents	6	2.9
Nonsexual assault	42	20.1
Nonsexual child abuse	51	24.4
Combat; war and terrorism	4	1.9
Witnessing death and injury	17	8.1
Other	13	6.2
Total	209	100

**TABLE 2:** Descriptives and correlations of study variables.

Variables	*n*	*M*	SD	1	2	3	4	5
1. PTSD severity	209	37.35	9.28	—				
2. AUD severity	209	15.67	12.40	−0.07	—			
3. DUD severity	209	18.06	13.46	0.25**	−0.29**	—		
4. Guilt	209	43.06	20.09	0.22**	−0.05	0.07	—	
5. Shame	209	31.47	16.72	0.43**	−0.04	0.15*	0.56**	—

*Note*: PTSD severity as measured by the CAPS‐5, AUD severity as measured by the AUDIT, DUD severity as measured by the DUDIT, Guilt as measured by the TRGI, and Shame as measured by the TRSI.

* *α* = 0.05.

** *α* = 0.01.

### Hypothesis 1: Associations between PTSD severity and SUD severity

PTSD severity was found to be a significant predicting factor of DUD severity. For AUD severity, no significant predictive effect of PTSD severity was found in a linear regression (see Table 3). Based on exploratory data analysis using scatterplots, we suspected a possible nonlinear relationship between PTSD severity and AUD severity. Analyses were rerun using squared *Z*‐scores to better capture this potential curvature. For AUD severity, PTSD severity was found to be a significant predicting factor in this exploratory regression (see Table 3).

**TABLE 3:** Associations between PTSD severity and SUD severity.

Dependent variables	*B* [95% CI]	*β*	*p*
AUD	0.01 [−0.18; 0.19]	0.005	0.940
DUD	0.34 [0.15; 0.52]	0.232	<0.001
AUD^a^	−0.99 [−1.97; −0.00]	−0.133	0.049
DUD^a^	0.10 [−0.95; 1.16]	0.013	0.846

*Note*: AUD as measured by the AUDIT, DUD as measured by the DUDIT, and PTSD as measured by the CAPS‐5.

^a^ Analysis performed with squared *Z*‐scores of the CAPS‐5.

### Hypothesis 2: Associations between trauma‐related shame and guilt and SUD severity

For both AUD severity and DUD severity, trauma‐related guilt was not found to be a significant predicting factor. Trauma‐related shame was found to be a significant predicting factor of DUD severity, but not of AUD severity (see Table 4).

**TABLE 4:** Associations between trauma‐related shame and guilt and SUD severity.

Dependent variables	Variables	*B* [95% CI]	*β*	*p*
AUD	Guilt	−0.02 [−0.10; 0.06]	−0.029	0.670
	Shame	0.00 [−0.10; 0.10]	0.003	0.961
DUD	Guilt	0.04 [−0.05; 0.12]	0.053	0.432
	Shame	0.11 [0.01; 0.22]	0.141	0.034

*Note*: AUD as measured by the AUDIT, DUD as measured by the DUDIT, Guilt as measured by the TRGI, and Shame as measured by the TRSI.

### Hypothesis 3: Associations between trauma‐related shame, guilt, and PTSD severity

Following the simple linear regressions, both trauma‐related guilt and shame were found to be significant predicting factors of PTSD severity. A multiple linear regression was used to test for a collective significant effect of trauma‐related guilt and shame on PTSD severity. The overall regression was statistically significant, *R* ^2^ = 0.185, *F*(2, 206) = 23.45, *p* < 0.001. When both trauma‐related guilt and trauma‐related shame were added as predictors in the same model, only trauma‐related shame independently predicted PTSD severity (see Table 5).

**TABLE 5:** Associations between trauma‐related shame, guilt, and PTSD severity.

Model	Variables	*B* [95% CI]	*β*	*p*
Model 1	Guilt	0.10 [0.03; 0.16]	0.205	0.003
Model 2	Shame	0.24 [0.17, 0.31]	0.420	<0.001
Model 3	Guilt	−0.02 [−0.09; 0.05]	−0.038	0.619
	Shame	0.25 [0.16; 0.33]	0.441	<0.001

*Note*: Guilt as measured by the TRGI and Shame as measured by the TRSI.

### Hypothesis 4: Associations between index trauma, trauma‐related shame, and PTSD severity

A one‐way ANOVA was performed to compare the effect of index trauma on trauma‐related shame. The results indicate a statistically significant difference in trauma‐related shame between at least two groups, *F*(7, 201) = 3.48, *p* = 0.002.

A one‐way ANOVA was performed to compare the effect of index trauma on PTSD severity. The results indicate no statistically significant differences in PTSD severity between at least two groups, *F*(7, 201) = 1.93, *p* = 0.067.

An exploratory one‐way ANOVA was performed to compare the effect of index trauma on trauma‐related guilt. The results indicate a statistically significant difference in trauma‐related guilt between at least two groups, *F*(7, 201) = 4.02, *p* < 0.001. See Appendix S1 on the rationale for this exploratory analysis.

#### Hypothesis 4a: Interpersonal versus noninterpersonal trauma

Results of the independent *t*‐tests showed a significant difference in trauma‐related shame between interpersonal and noninterpersonal trauma. For PTSD severity, no significant difference was found. For trauma‐related guilt, a significant difference was found (see Table 6).

**TABLE 6:** Differences in trauma‐related shame and PTSD severity between interpersonal and noninterpersonal traumas.

Dependent variables	Interpersonal (*n* = 169)		Noninterpersonal (*n* = 40)		*t*	*p*	Cohen's *d*
	*M*	SD	*M*	SD			
Guilt	45.16	18.39	34.98	19.44	3.115	0.002	0.55
Shame	33.50	16.18	22.88	16.41	3.725	<0.001	0.66
PTSD severity	37.63	9.64	36.18	7.57	0.893	0.373	0.16

*Note*: Shame as measured by the TRSI, PTSD severity as measured by the CAPS‐5, and Guilt as measured by the TRGI.

#### Hypothesis 4b: Sexual versus physical interpersonal trauma

Results of the independent *t*‐tests showed a significant difference in trauma‐related shame between sexual and physical interpersonal trauma. For PTSD severity, no significant difference was found. For trauma‐related guilt, a significant difference was found (see Table 7).

**TABLE 7:** Differences in trauma‐related shame and PTSD severity between sexual and physical interpersonal traumas.

Dependent variables	Sexual trauma (*n* = 76)		Physical trauma (*n* = 93)		*t*	*p*	Cohen's *d*
	*M*	SD	*M*	SD			
Guilt	50.25	17.93	41.00	17.81	3.349	0.001	0.52
Shame	36.47	15.66	31.08	16.27	2.181	0.031	0.34
PTSD severity	38.66	10.09	36.80	9.23	1.251	0.213	0.19

*Note*: Shame as measured by the TRSI, PTSD severity as measured by the CAPS‐5, and Guilt as measured by the TRGI.

#### Hypothesis 4c: Childhood versus nonchildhood interpersonal trauma

Results of the independent *t*‐tests showed no significant difference in trauma‐related shame between childhood and nonchildhood interpersonal trauma. A significant difference in PTSD severity was found. No significant difference in trauma‐related guilt was found (see Table 8).

**TABLE 8:** Differences in trauma‐related shame and PTSD severity between childhood and nonchildhood interpersonal traumas.

Dependent variable	Childhood trauma (*n* = 106)		Nonchildhood trauma (*n* = 63)		*t*	*p*	Cohen's *d*
	*M*	SD	*M*	SD			
Guilt	43.35	18.84	48.21	17.34	−1.669	0.097	−0.27
Shame	34.54	16.06	31.76	16.37	1.079	0.282	0.17
PTSD severity	39.31	10.08	34.81	8.18	3.004	0.003	0.48

*Note*: Shame as measured by the TRSI, PTSD severity as measured by the CAPS‐5, and Guilt as measured by the TRGI.

#### Hypothesis 4d: Moderating effect of index trauma on the association between trauma‐related shame and PTSD severity

Moderation analysis was conducted using SPSS's PROCESS macro (Hayes, 2013) to test for a possible moderating effect of index trauma (childhood vs. nonchildhood) on the relationship between trauma‐related shame and PTSD severity. As expected, the overall model was significant, *F*(3, 165) = 17.74, *p* < 0.001, *R* ^2^ = 0.24. The interaction between trauma‐related shame (*X*) and index trauma (*W*) was not significant (*b* = −0.08, SE = 0.08, *t*(3) = −0.99, *p* = 0.322), indicating that the relationship between trauma‐related shame (*X*) and PTSD severity (*Y*) was not moderated by index trauma (*W*).

## DISCUSSION

The present study aimed to examine the associations between PTSD severity, SUD severity, trauma‐related shame, trauma‐related guilt, and type of index trauma in a treatment‐seeking co‐occurring PTSD and SUD population.

First, we examined the predictive value of PTSD severity on SUD severity. In line with our hypothesis, PTSD severity was found to be a significant predictor of DUD severity. For AUD severity, no linear association with PTSD severity was found, but a curvilinear association was found. Our findings indicate that for DUD severity, more severe PTSD symptoms are associated with more severe drug use problems. This aligns with previous research on the high co‐occurrence of PTSD and SUD (Driessen et al., 2008; Pietrzak et al., 2011) and the association between the two disorders (Berenz et al., 2017). The relationship between PTSD symptoms and AUD severity follows a similar pattern to that of DUD severity up to a certain threshold. Beyond this point, the relationship seems to shift, with more severe PTSD symptoms predicting less severe AUD severity. One explanation for this curvilinear relationship could be that self‐medication of PTSD symptoms through alcohol use (as proposed by Khantzian, 1985) is effective for moderately severe PTSD symptoms but becomes insufficient as the severity of PTSD symptoms increases. Alternatively, self‐medication through alcohol use may initially be effective in managing PTSD symptoms without leading to significant alcohol‐related problems. However, over time, untreated AUD exacerbates (McCabe et al., 2022), potentially altering social support and psychological resources, which could, in turn, affect the severity of PTSD symptoms. We recommend that future research involving patients with co‐occurring PTSD and SUD further investigate the predictive value of PTSD severity on SUD severity to facilitate comparison with the findings presented in this study.

Second, we examined the predictive value of trauma‐related shame and trauma‐related guilt on SUD severity. Our findings on trauma‐related guilt are in line with the findings of Capone et al. (2020) and suggest that trauma‐related guilt is not a significant predictor of either AUD or DUD severity. Partially in line with our hypothesis, trauma‐related shame was significantly associated with DUD severity but not with AUD severity. This finding on DUD severity aligns with the findings of Saraiya et al. (2024) on the prospective association between trauma‐related shame and opioid use. Replication of our findings is needed in order to conclude whether trauma‐related shame is more strongly linked to drug use disorders than alcohol use disorders. One explanation for not finding the association of trauma‐related shame with AUD severity could be that we based our hypothesis on literature mostly focusing on shame proneness rather than trauma‐related shame as it is gauged by the TRSI (Holl et al., 2017; Luoma et al., 2017, 2019). This difference between trauma‐related shame and shame proneness and their relations to SUD has not received considerable attention in previous literature. Thus, replication of our data is needed in order to conclude whether shame proneness and trauma‐related shame are different in their relation to SUD (or AUD specifically). Alternatively, it might be that the association between trauma‐related shame and AUD severity disappears over time. A previous meta‐analysis found shame to be related to SUD severity when assessed in recently developed SUD but not when assessed in long‐existing SUD (Luoma et al., 2019; mean years of SUD in our sample = 12.21 years). More research into the association between specific forms of shame and SUD severity should be conducted in patient groups with co‐occurring PTSD and SUD to find consensus on this topic.

Third, we examined the predictive value of trauma‐related shame and trauma‐related guilt on PTSD severity. As hypothesized, both trauma‐related shame and guilt are significantly associated with PTSD severity. In addition, as expected, in the multiple model, only trauma‐related shame was significantly associated with PTSD severity. The effect of trauma‐related guilt was canceled out by the addition of trauma‐related shame into the model. Although some previous studies also found a direct relation between trauma‐related guilt and PTSD severity (Held et al., 2011), more evidence points to the direction of an effect of trauma‐related guilt through trauma‐related shame on PTSD severity (Held et al., 2011, 2015), which is in line with our results. This effect is explained by the model of Kubany and Watson (2003), which suggests that trauma‐related guilt is indirectly associated with psychological distress through disengagement or avoidant coping. However, since the construct of trauma‐related guilt is conceptualized differently across several studies, it is more difficult to draw general conclusions about the impact of trauma‐related guilt (Wiseman et al., 2021). Furthermore, our findings align with prior research indicating that trauma‐related shame is robustly associated with PTSD severity (Badour et al., 2017; López‐Castro et al., 2019) and with studies that found trauma‐related shame to be more pathogenic than trauma‐related guilt (Leskela et al., 2002; Saraiya & Lopez‐Castro, 2016). An explanation for the relation between trauma‐related shame and PTSD severity could be that shame is significantly correlated to autonomic arousal during exposure to trauma triggers (Freed & D'Andrea, 2015). As a result, trauma‐related shame is an important emotion and a possible focus point for the treatment of PTSD (Held et al., 2015), not only in a PTSD‐only population but also in a population with co‐occurring PTSD–SUD. Treating shame can possibly improve treatment completion, handle avoidance symptoms, and lead to better outcomes of PTSD treatment (Saraiya & Lopez‐Castro, 2016). However, no conclusions about the causality between trauma‐related shame and PTSD severity can be drawn based upon our study findings.

Fourth, we examined the association between the type of index trauma and trauma‐related shame, PTSD severity, and trauma‐related guilt, respectively. In line with our hypothesis, both interpersonal trauma and sexual trauma were associated with more trauma‐related shame compared with noninterpersonal trauma and physical trauma, respectively. We found the same results for trauma‐related guilt. In regard to PTSD severity, both interpersonal trauma and sexual trauma were not associated with more PTSD severity compared with noninterpersonal trauma and physical trauma, respectively. When childhood interpersonal trauma and nonchildhood interpersonal trauma were compared, childhood interpersonal trauma was significantly more related to PTSD severity but not to trauma‐related shame or trauma‐related guilt. Taken together, these results suggest that the type of index trauma does affect trauma‐related shame, PTSD severity, and trauma‐related guilt; however, in different ways: for trauma‐related shame and guilt, the subject of the type of index trauma appears to be relevant, and the onset (childhood vs. nonchildhood) does not, whereas for PTSD severity, the onset (childhood vs. nonchildhood) appears to be more relevant, and the subject of the index trauma does not. Following the research of Dunn et al. (2017), it might be worthwhile for future research to focus on the differing effects of age at trauma exposure (e.g., early childhood, middle childhood, or adolescence) on trauma‐related shame and PTSD severity. Possibly, exposure to traumatic events in early childhood is more pathogenic than exposure to traumatic events later in childhood. We did not account for this distinction in age at trauma exposure in our study. Still, our results suggest that early detection of complaints is especially important to prevent the development of severe PTSD symptoms. Moreover, considering shame as a focus point for PTSD treatment, it becomes crucial for individuals who have experienced interpersonal and especially sexual interpersonal trauma, regardless of when the trauma occurred. Since the type of index trauma was associated with PTSD severity, we explored whether index trauma (childhood vs. nonchildhood) moderated the relation between trauma‐related shame and PTSD severity. This was not the case. Again, it might be possible for future research to find a moderating effect of age on the association between trauma‐related shame and PTSD severity if more differences in age could be accounted for.

### Strengths and limitations

This study has several strong points. First, most studies about associations between PTSD and SUD used nonclinical samples, whereas our sample consisted of a group of patients with both disorders. Second, we used two separate measures for trauma‐related guilt and trauma‐related shame instead of one questionnaire, as has been done in several previous studies (Woien et al., 2003). This allowed us to examine the two measures independently of one another and thus more accurately.

This study also has some limitations. First, this is a SUD‐treatment‐seeking sample of patients with co‐occurring SUD and PTSD, making it difficult to generalize results to all patients with both disorders. Possibly, PTSD‐treatment‐seeking patients with co‐occurring PTSD and SUD would show different results since their PTSD severity might be higher and their SUD severity might be lower. Second, in response to the distinction between guilt and shame proneness and trauma‐related guilt and shame, this study did not measure guilt and shame proneness, and therefore, we could not control for an individual's proneness to guilt and shame. This would make for a good addition for future research. Finally, this study did not differentiate between single versus multiple traumas. Therefore, we could not examine whether the effect of index trauma could also be explained by the experience of multiple trauma (Hagenaars et al., 2011).

## CONCLUSION

Our study did find associations between PTSD severity and SUD severity and trauma‐related shame and DUD severity. In addition, our study did yield evidence for a notable association between PTSD severity and, in particular, trauma‐related shame in patients with SUD and PTSD. However, when it comes to index trauma characteristics, trauma‐related shame and PTSD severity show two different patterns. For trauma‐related shame, the trauma subject appears to be related (interpersonal and sexual trauma), whereas for PTSD severity, the age of onset appears to be related. Taken together, these results suggest that it is important to specifically ask patients about trauma‐related shame in PTSD treatment, especially when the patient has a history of interpersonal and/or sexual abuse.

## FUNDING INFORMATION

This work was funded by The Dutch “Stichting tot steun VCVGZ” who assigned an investment subsidy to Prof. dr. Arnoud Arntz at the University of Amsterdam [grant numbers 244]. The funding source had no role in the design of the study and has no authority over the conduct of the study; collection, analysis, and interpretation of the data; preparation, review or approval of the manuscript; or the decision to submit the manuscript for publication.

## CONFLICT OF INTEREST STATEMENT

The authors declare that they have no competing interests.

## ETHICS STATEMENT

Ethics approval and consent to participate in the TOPA study were given by the Medical Ethical Committee of the Amsterdam Academic Medical Centre.

## PATIENT CONSENT STATEMENT

Written informed consent from the participants is obtained during the screening process prior to the first assessment.

## Supporting information


Appendix S1

## Data Availability

The study reported in this article was not formally preregistered. Since the research group is also writing other articles about this data, the raw data remain confidential at this point but will become available after other articles are published from the corresponding author on reasonable request.
