# Supplementary material for: Trauma‐related guilt, shame, and trauma type among patients with co‐occurring PTSD and SUD
Source: Alcohol Clin Exp Res (Hoboken). 2025 Mar 26;49(5):1064–75. doi: 10.1111/acer.70028 (PMC12098809; doi:10.1111/acer.70028)
Supplement: Supplementary file 1 — Appendix S1 [file ACER-49-1064-s001.docx]

**Supplementary information**

Regarding the TRGI: Based on the suggestion of an anonymous reviewer, we opted to use the Guilt Cognitions scale. However, we also conducted the analyses using the subscale specified in the study protocol (Lortye et al., 2021). This subscale consists of all items of the TRGI, minus the items belonging to the Guilt Distress scale and the Lack of Justification scale, in line with research of Boterhoven-de Haan et al. (2020). The results were consistent across both approaches.

Regarding analyses on SUD-severity: Initially, regression analyses were performed for the AUDIT and the DUDIT separately, for which only the data of participants with a primary diagnosis of alcohol use disorder were used for analyses with the AUDIT (N = 99) and only the data of participants with a primary diagnosis on another substance use disorder were used for analyses with the DUDIT (N = 110). All associations (PTSD severity, trauma-related guilt, and trauma-related shame) were non-significant. Based on a suggestion of an anonymous reviewer, we have opted to re-examine the regression analyses on the total group, as this approach provides greater statistical power and increases the variability in AUDIT and DUDIT scores.

Regarding the supplementary analyses performed with trauma-related guilt for hypothesis 4: On request of anonymous reviewers, the associations between index trauma and trauma-related guilt were explored.

Regarding the analyses performed with covariates included, we provide the results in this supplementary file. See Tables S1–S3 below.

**Table S1**

*Associations between PTSD-severity and SUD-severity, including covariates*

| Dependent variable | Independent variable | B [95% CI] | β | *p* |
| --- | --- | --- | --- | --- |
| AUD | PTSD | .04 [-0.15; 0.22] | .028 | .690 |
|  | Location | -1.07 [-4.57; 2.44] | -.041 | .548 |
|  | Sex | -0.37 [-3.70; 2.97] | -.015 | .829 |
|  | Age^1^ | 0.164 [0.02; 0.31] | .159 | .024 |
|  | Duration of PTSD symptoms^1^ | 0.03 [-0.13; 0.19] | .034 | .699 |
|  | Duration of SUD symptoms^1^ | 0.09 [-0.10; 0.28] | .077 | .357 |
| DUD | PTSD | 0.29 [0.11; 0.48] | .202 | .002 |
|  | Location | 2.26 [-1.41; 5.93] | .079 | .225 |
|  | Sex | -2.60 [-6.09; 0.88] | -.097 | .142 |
|  | Age^1^ | -0.16 [-0.31; -0.01] | -.140 | .040 |
|  | Duration of PTSD symptoms^1^ | -0.01 [-0.14; 0.12] | -.007 | .919 |
|  | Duration of SUD symptoms^1^ | 0.11 [-0.06; 0.27] | .084 | .214 |

*Note.* AUD as measured by the AUDIT, DUD as measured by the DUDIT, PTSD as measured by the CAPS-5. ^1^Age and Duration of symptoms (PTSD/SUD) were run in separate analyses due to high intercorrelation, which resulted in minor decimal changes in the coefficients of other covariates.

**Table S2**

*Associations between trauma-related shame and guilt and SUD-severity, including covariates*

| Dependent variable | Variable | B [95% CI] | β | *p* |
| --- | --- | --- | --- | --- |
| AUD | Guilt | -.01 [-0.09; 0.08] | -.008 | .911 |
|  | Location | -0.96 [-4.45; 2.53] | -.037 | .587 |
|  | Sex | -0.33 [-3.68; 3.03] | -.013 | .847 |
|  | Age | 0.16 [0.02; 0.30] | .155 | .027 |
|  | Duration of PTSD symptoms^1^ | 0.11 [-0.01; 0.24] | .121 | .075 |
|  | Duration of SUD symptoms^1^ | 0.16 [0.01; 0.32] | .138 | .043 |
|  | Shame | .00 [-0.10; 0.10] | .003 | .961 |
|  | Location | -0.98 [-4.46; 2.50] | -.037 | .579 |
|  | Sex | -0.35 [-3.69; 2.99] | -.014 | .835 |
|  | Age | 0.16 [0.02; 0.30] | .156 | .026 |
|  | Duration of PTSD symptoms^1^ | 0.11 [-0.01;0.23] | .120 | .076 |
|  | Duration of SUD symptoms^1^ | 0.16 [0.01; 0.32] | .139 | .042 |
| DUD | Guilt | 0.03 [-0.06; 0.11] | .037 | .577 |
|  | Location | 3.03 [-0.69; 6.76] | .106 | .110 |
|  | Sex | -2.68 [-6.26; 0.90] | -.100 | .141 |
|  | Age | -0.18 [-0.34; -0.03] | -.164 | .018 |
|  | Duration of PTSD symptoms^1^ | -0.00 [-0.14; 0.13] | -.003 | .960 |
|  | Duration of SUD symptoms^1^ | 0.08 [-0.09; 0.25] | .066 | .338 |
|  | Shame | 0.11 [0.01; 0.22] | .139 | .033 |
|  | Location | 2.97 [-0.71; 6.64] | .104 | .113 |
|  | Sex | -2.74 [-6.27; 0.79] | -.102 | .127 |
|  | Age | -0.19 [-0.34; -0.04] | -.167 | .015 |
|  | Duration of PTSD symptoms^1^ | -0.02 [-0.15; 0.12] | -.018 | .794 |
|  | Duration of SUD symptoms^1^ | 0.08 [-0.09; 0.25] | .064 | .346 |

*Note.* AUD as measured by the AUDIT, DUD as measured by the DUDIT, Guilt as measured by the TRGI, and Shame as measured by the TRSI. ^1^Age and Duration of symptoms (PTSD/SUD) were run in separate analyses due to high intercorrelation, which resulted in minor decimal changes in the coefficients of other covariates.

**Table S3**

*Associations between trauma-related shame, guilt and PTSD-severity, including covariates*

| Variable | B [95% CI] | β | *p* |
| --- | --- | --- | --- |
| Guilt | -0.04 [-0.10; 0.04] | -.075 | .326 |
| Shame | 0.25 [0.17; 0.34] | .450 | <.001 |
| Location | 2.70 [0.27; 5.14] | .138 | .030 |
| Sex | -0.02 [-2.38; 2.33] | -.001 | .985 |
| Age | -0.11 [-0.21; -0.02] | -.147 | .023 |
| Duration of PTSD symptoms^1^ | -0.04 [-0.12; 0.05] | -.050 | .440 |
| Duration of SUD symptoms^1^ | -0.02 [-0.15; 0.12] | -.018 | .818 |

*Note.* Guilt as measured by the TRGI, and Shame as measured by the TRSI. ^1^Age and Duration of symptoms (PTSD/SUD) were run in separate analyses due to high intercorrelation, which resulted in minor decimal changes in the coefficients of other covariates.
